# Metagenome-Assembled Genomes from a Microbiome Grown in Dairy Manure Hydrolysate

**DOI:** 10.1128/mra.00292-22

**Published:** 2022-07-27

**Authors:** Abel T. Ingle, Nathaniel W. Fortney, Kevin S. Myers, Kevin A. Walters, Matthew J. Scarborough, Timothy J. Donohue, Daniel R. Noguera

**Affiliations:** a Great Lakes Bioenergy Research Center, University of Wisconsin–Madison, Madison, Wisconsin, USA; b Wisconsin Energy Institute, University of Wisconsin–Madison, Madison, Wisconsin, USA; c Department of Civil and Environmental Engineering, University of Wisconsin–Madison, Madison, Wisconsin, USA; d Department of Bacteriology, University of Wisconsin–Madison, Madison, Wisconsin, USA; e Department of Civil and Environmental Engineering, University of Vermont, Burlington, Vermont, USA; Indiana University, Bloomington

## Abstract

Anaerobic microbiomes can be used to recover the chemical energy in agroindustrial and municipal wastes as useful products. Here, we report a total of 109 draft metagenome-assembled genomes from a bioreactor-fed carbohydrate-rich dairy manure hydrolysate. Studying these genomes will aid us in deciphering the metabolic networks in anaerobic microbiomes.

## ANNOUNCEMENT

**FIG 1:**
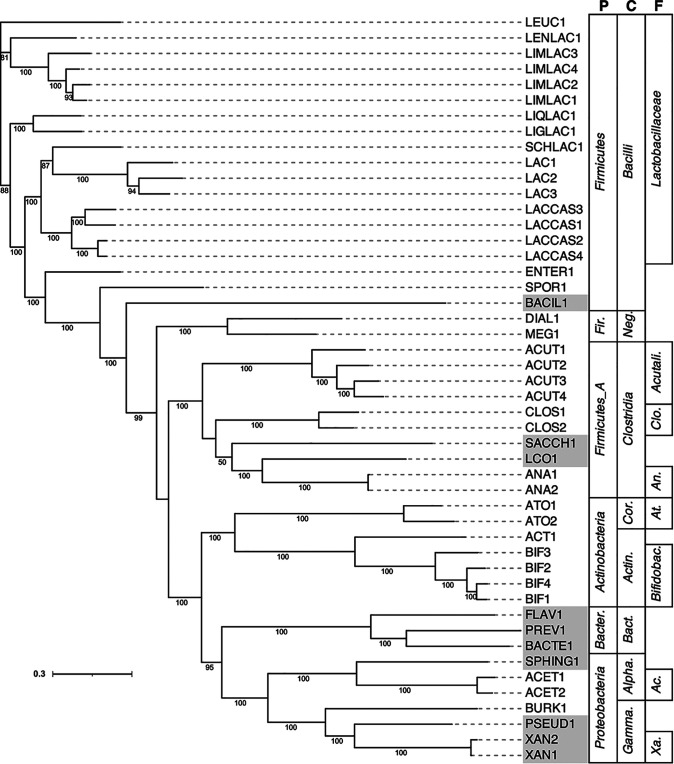
Phylogeny of dRep-identified representative MAGs. Strain code abbreviations are as follows: ACET, *Acetobacter*; ATO, *Atopobiaceae*; ACT, *Actinomycetaceae*; ACUT, *Acutalibacteraceae*; ANA, *Anaerotignaceae*; BAC, *Bacillus*; BACIL, *Bacilli*; BACTE, *Bacteroidales*; BIF, *Bifidobacteriaceae*; BURK, *Burkholderiaceae*; CLOS, *Clostridium*; DIAL, *Dialister*; ENTER, *Enterococcaceae*; FLAV, *Flavobacteriales*; LAC, *Lactobacillus*; LACCAS, *Lacticaseibacillus*; LCO, *Lachnospiraceae*; LENLAC, *Lentilactobacillus*; LEUC, *Leuconostoc*; LIMLAC, *Limosilactobacillus*; LIGLAC, *Ligilactobacillus*; LIQLAC, *Liquorilactobacillus*; MEG, *Megasphaera*; PREV, *Prevotella*; PSEUD, Pseudomonas; SACCH, *Saccharofermentans*; SCHLAC, *Schleiferilactobacillus*; SPHING, *Sphingobium*; SPOR, *Sporolactobacillus*; XAN, *Xanthomonadaceae*. The higher taxonomic levels are labeled P, phylum; C, class; and F, family. Phylum abbreviations: *Bacter*., *Bacteroidota*; *Fir*., *Firmicutes_C*. Class abbreviations: *Gamma*., *Gammaproteobacteria*; *Alpha*., *Alphaproteobacteria*; *Bact*., *Bacteroidia*; *Actin*., *Actinobacteria*; *Cor*., *Coriobacteriia*; *Neg*., *Negativicutes*. Family abbreviations: *Xa*., *Xanthomonadaceae*; *Ac*., *Acetobacter*; *Bifidobac.*, *Bifidobacteriaceae*; *At*., *Atopobiaceae*; *An*., *Anaerotignaceae*; *Clo*., *Clostridiaceae*; *Acutali*., *Acutalibacteraceae*. The shaded strain code abbreviations indicate representative genomes from the inoculum. The phylogenetic tree was generated in RAxML-NG with 500 bootstraps using the concatenation of 120 bacterial single-copy housekeeping genes generated using GTDB-Tk. Bootstrap values greater than 50 are shown. The scale bar indicates the number of nucleotide substitutions per sequence site.

We are investigating microbial fermentation to valorize agroindustrial residues (1–5). Previously, we reported on the fermentation product profile produced when feeding dairy manure (DM) hydrolysate to an anaerobic bioreactor (3), and here, we report on the microbial community. The bioreactor was inoculated with acid-phase anaerobic digester sludge from the Nine Springs Wastewater Treatment Plant (Madison, WI, USA) (2–5). DNA was extracted from the inoculum and at multiple time points during bioreactor operation using a phenol-chloroform extraction method (4) that excluded a bead-beating step so that the DNA fragment lengths were appropriate for long-read sequencing. The DNA quantity and quality were determined using a Qubit v4.0 fluorometer (Thermo Fisher Scientific, Waltham, MA, USA) and a NanoDrop ND-1000 spectrophotometer (Thermo Fisher Scientific), respectively. DNA aliquots of 500 ng (6 samples, all from the bioreactor) and 3,000 ng (4 samples, 2 from the bioreactor and 2 from the inoculum) were submitted to the Joint Genome Institute (JGI; https://jgi.doe.gov/; Berkeley, CA, USA) for paired-end 2 × 150-bp sequencing on the NovaSeq S4 platform (Illumina, Inc., San Diego, CA, USA) and long-read sequencing using the PacBio Sequel II platform (Pacific Biosciences, Inc., Menlo Park, CA, USA), respectively. The Illumina libraries were end repaired, A-tailed, and ligated with Illumina-compatible adapters using the KAPA HyperPrep kit (Roche, USA) as described (6). The PacBio sequencing library preparation included shearing genomic DNA (Blue Pippin size selection; Sage Science, USA) to 6 to 10 kb and performing ligation using the SMRTbell Express template prep kit v2.0 following the manufacturer’s protocol (Pacific Biosciences). All software was used with default parameters unless otherwise noted. The Illumina reads were filtered and error corrected using bbcms from BBMap v38.86 (mincount = 2, highcountfraction = 0.6) (7), assembled using metaSPAdes v3.14.1 (8), and mapped using BBMap v38.86 (ambiguous=random) (7) following the JGI metagenomic workflow (6). The PacBio circular consensus sequencing (CCS) reads were assembled using metaFlye v2.8.1-b1676 (–meta) (9), polished using GCpp v1.0.0-SL-release-8.0.0 (Pacific Biosciences), mapped using minimap2 v2.12-r941 (10), and binned using MetaBAT v2:2.15 (11). The resulting Illumina metagenomic libraries contained between 78 and 114 million reads with a targeted length of 150 bp, and the PacBio libraries contained between 132,000 and 537,000 CCS reads with a targeted length of 6 to 10 kb. The resulting metagenome-assembled genomes (MAGs) were annotated using the JGI annotation pipeline v5.0.23 (12) and the NCBI Prokaryotic Genome Annotation Pipeline v6.0 (13). The MAGs were then refined by removing contigs deemed to be contaminants using ProDeGe v2.3 (14) and custom scripts that compare tetranucleotide frequency among contigs (run.GC.sh and Calculating_TF_Correlations.R; https://github.com/GLBRC/metagenome_analysis). All refined MAGs were dereplicated into clusters (i.e., replicate genome sets) using dRep v3.2.2 (15). The MAG quality parameters were obtained using CheckM v1.0.11 (16), and taxonomy was assigned using GTDB-Tk v1.5.1 (database release 202) (17). The MAG phylogeny was visualized using RAxML-NG v0.9.0 (Fig. 1) (18) and Interactive Tree of Life v5 (19).

We provide a resource containing a total of 109 MAGs with greater than 75% completion and less than 3% contamination, grouped into 48 clusters that represent the microbial community diversity (Table 1). The putative classifications of these clusters span the phyla *Firmicutes*, *Actinobacteria*, *Bacteroidota*, and *Proteobacteria* (Fig. 1).

**TABLE 1:** Statistics and accession numbers of refined metagenome-assembled genomes

Code^*a*^	Strain name^*b*^	Sample source^*c*^	GenBank accession no.	SRA accession no.^*d*^	ANIm^*e*^	dRep^*f*^	GTDB-Tk classification^*g*^	Reference genome GenBank accession no.^*h*^	Sequencing platform	Completeness (%)	Contamination (%)	MAG size (Mbp)	No. of scaffolds	*N*_50_ (Mbp)	%GC	No. of tRNAs	No. of rRNAs:		
																	5S	16S	23S
ACET1	UW_DM_ACET1_1	DMB	JALCOP000000000.1	SRX12654472		124.3612	d__Bacteria;p__Proteobacteria;c__Alphaproteobacteria;o__Acetobacterales;f__Acetobacteraceae;g__Acetobacter;s__Acetobacter fabarum	GCF_011516925.1	Illumina NovaSeq S4	95.61	0.25	2.324	10	0.593	58.95	39	0	0	0
	UW_DM_ACET1_2	DMB	JALCRJ000000000.1	SRX12654480	0.999971	123.3789			Illumina NovaSeq S4	95.85	0.25	2.372	10	0.338	58.86	39	0	0	0
	UW_DM_ACET1_3	DMB	JALCPS000000000.1	SRX12654476	0.999977	123.3504			Illumina NovaSeq S4	95.85	0.25	2.328	9	0.333	58.92	39	0	0	0
	UW_DM_ACET1_4	DMB	JALCQH000000000.1	SRX12654477	0.999979	123.3504			Illumina NovaSeq S4	95.85	0.25	2.323	10	0.333	58.95	39	0	0	0
	UW_DM_ACET1_5	DMB	JALCPA000000000.1	SRX12654474	0.999992	123.2260			Illumina NovaSeq S4	95.85	0.25	2.323	11	0.315	58.95	39	0	0	0
ACET2	UW_DM_ACET2_1	DMB	JALCRI000000000.1	SRX12654480		121.2990	d__Bacteria;p__Proteobacteria;c__Alphaproteobacteria;o__Acetobacterales;f__Acetobacteraceae;g__Acetobacter;s__Acetobacter peroxydans	GCF_006539345.1	Illumina NovaSeq S4	97.01	0.03	2.422	43	0.072	60.81	43	0	0	0
	UW_DM_ACET2_2	DMB	JALCQV000000000.1	SRX12654475	0.999847	119.2871			Illumina NovaSeq S4	96.18	0.5	2.277	77	0.037	61.1	40	0	0	0
ACT1	UW_DM_ACT1_1	DMB	JALCPR000000000.1	SRX12654476		126.2383	d__Bacteria;p__Actinobacteriota;c__Actinomycetia;o__Actinomycetales;f__Actinomycetaceae;g__Ancrocorticia;s__	NA	Illumina NovaSeq S4	98.32	0	2.285	15	0.382	58.24	48	0	0	0
	UW_DM_ACT1_2	DMB	JALCQG000000000.1	SRX12654477	0.999918	125.5519			Illumina NovaSeq S4	99.16	0	2.157	21	0.189	58.11	48	1	0	0
	UW_DM_ACT1_3	DMB	JALCRH000000000.1	SRX12654480	0.999083	121.0378			Illumina NovaSeq S4	97.48	0	1.936	53	0.051	58.23	44	0	0	0
	UW_DM_ACT1_4	DMB	JALCQU000000000.1	SRX12654475	0.999271	120.4459			Illumina NovaSeq S4	96.64	0	1.872	48	0.057	58.31	40	0	0	0
	UW_DM_ACT1_5	DMB	JALCOZ000000000.1	SRX12654474	0.997915	116.1328			Illumina NovaSeq S4	92.44	0	1.773	54	0.055	58.37	37	1	0	0
	UW_DM_ACT1_6	DMB	JALCOO000000000.1	SRX12654472	0.998142	114.4529			Illumina NovaSeq S4	90.76	0	1.717	49	0.055	58.44	36	0	0	0
ACUT1	UW_DM_ACUT1_1	DMB	JALCRG000000000.1	SRX12654480		125.8525	d__Bacteria;p__Firmicutes_A;c__Clostridia;o__Oscillospirales;f__Acutalibacteraceae;g__UBA4871;s__UBA4871 sp902809935	GCF_902809935.1	Illumina NovaSeq S4	97.85	0.34	2.301	6	0.429	42.76	47	3	0	0
	UW_DM_ACUT1_2	DMB	JALCQF000000000.1	SRX12654477	0.999982	125.8261			Illumina NovaSeq S4	97.85	0.34	2.327	7	0.424	42.74	47	3	0	0
	UW_DM_ACUT1_3	DMB	JALCQT000000000.1	SRX12654475	1.000000	125.8261			Illumina NovaSeq S4	97.85	0.34	2.292	6	0.424	42.76	47	3	0	0
	UW_DM_ACUT1_4	DMB	JALCPQ000000000.1	SRX12654476	0.999961	125.8261			Illumina NovaSeq S4	97.85	0.34	2.328	7	0.424	42.74	47	3	0	0
ACUT2	UW_DM_ACUT2_1	DMB	JALCPP000000000.1	SRX12654476		123.9766	d__Bacteria;p__Firmicutes_A;c__Clostridia;o__Oscillospirales;f__Acutalibacteraceae;g__Caprobacter;s__	NA	Illumina NovaSeq S4	97.99	0.02	2.551	18	0.250	48.65	46	2	0	1
ACUT3	UW_DM_ACUT3_1	DMB	JALCPO000000000.1	SRX12654476		116.5123	d__Bacteria;p__Firmicutes_A;c__Clostridia;o__Oscillospirales;f__Acutalibacteraceae;g__Caprobacter;s__	NA	Illumina NovaSeq S4	94.07	0.67	2.111	81	0.036	58.11	34	1	1	0
	UW_DM_ACUT3_2	DMB	JALCRF000000000.1	SRX12654480	0.999724	114.4897			Illumina NovaSeq S4	92.11	0.67	2.200	77	0.035	58.02	41	1	0	0
	UW_DM_ACUT3_3	DMB	JALCQS000000000.1	SRX12654475	0.999801	101.0366			Illumina NovaSeq S4	78.91	0	1.933	88	0.027	58.05	31	1	0	0
ACUT4	UW_DM_ACUT4_1	DMB	JALCQE000000000.1	SRX12654477		102.1662	d__Bacteria;p__Firmicutes_A;c__Clostridia;o__Oscillospirales;f__Acutalibacteraceae;g__Caprobacter;s__Caprobacter sp002407675	GCA_002407675.1	Illumina NovaSeq S4	80.54	0.67	1.727	52	0.039	52.04	24	1	0	0
ANA1	UW_DM_ANA1_1	DMB	JALCQR000000000.1	SRX12654475		123.9165	d__Bacteria;p__Firmicutes_A;c__Clostridia;o__Lachnospirales;f__Anaerotignaceae;g__HGM11808;s__	NA	Illumina NovaSeq S4	97.99	0.89	2.610	19	0.187	36.68	41	1	2	1
	UW_DM_ANA1_2	DMB	JALCRE000000000.1	SRX12654480	0.999999	122.4693			Illumina NovaSeq S4	96.64	0.89	2.541	20	0.179	36.5	33	0	2	0
	UW_DM_ANA1_3	DMB	JALCOY000000000.1	SRX12654474	0.999702	102.0096			Illumina NovaSeq S4	80.35	1.57	2.007	92	0.024	36.32	19	0	2	0
ANA2	UW_DM_ANA2_1	DMB	JALCPN000000000.1	SRX12654476		98.4501	d__Bacteria;p__Firmicutes_A;c__Clostridia;o__Lachnospirales;f__Anaerotignaceae;g__HGM11808;s__	NA	Illumina NovaSeq S4	77.08	0.56	2.332	94	0.030	36.21	26	0	1	1
ATO1	UW_DM_ATO1_1	DMB	JALCQQ000000000.1	SRX12654475		125.7981	d__Bacteria;p__Actinobacteriota;c__Coriobacteriia;o__Coriobacteriales;f__Atopobiaceae;g__Olsenella;s__Olsenella sp900555995	GCF_009695875.1	Illumina NovaSeq S4	99.8	0.08	2.182	19	0.254	65	50	0	0	0
ATO2	UW_DM_ATO2_1	DMB	JALCPM000000000.1	SRX12654476		121.6595	d__Bacteria;p__Actinobacteriota;c__Coriobacteriia;o__Coriobacteriales;f__Atopobiaceae;g__Olegusella;s__Olegusella sp002407925	GCA_002407925.1	Illumina NovaSeq S4	94.89	0	1.610	9	0.356	53.87	46	1	1	1
BACIL1	UW_DM_BACIL1_1	Inoc.	JALCLG000000000.1	SRR19542533, SRR19542534		127.2301	d__Bacteria;p__Firmicutes;c__Bacilli;o__RFN20;f__CAG-288;g__UBA7642;s__	NA	PacBio Sequel II	96.63	0	2.080	1	2.080	49.99	48	2	2	2
BACTE1	UW_DM_BACTE1_1	Inoc.	JALCKZ000000000.1	SRR19542531, SRR19542532		127.3472	d__Bacteria;p__Bacteroidota;c__Bacteroidia;o__Bacteroidales;f__UBA932;g__UBA1232;s__	NA	PacBio Sequel II	95.84	0.48	2.229	1	2.229	40.66	40	2	2	2
	UW_DM_BACTE1_2	Inoc.	JALCLF000000000.1	SRR19542533, SRR19542534	0.996672	107.4680			PacBio Sequel II	77.25	0.48	1.692	2	1.231	40.66	30	2	2	2
BIF1	UW_DM_BIF1_1	DMB	JALCQD000000000.1	SRX12654477		128.1029	d__Bacteria;p__Actinobacteriota;c__Actinomycetia;o__Actinomycetales;f__Bifidobacteriaceae;g__;s__	NA	Illumina NovaSeq S4	100	0	2.162	6	0.659	57.32	51	2	0	0
BIF2	UW_DM_BIF2_1	DMB	JALCQC000000000.1	SRX12654477		120.0066	d__Bacteria;p__Actinobacteriota;c__Actinomycetia;o__Actinomycetales;f__Bifidobacteriaceae;g__;s__	NA	Illumina NovaSeq S4	94.86	0	2.059	32	0.107	57.09	45	0	0	0
	UW_DM_BIF2_2	DMB	JALCPL000000000.1	SRX12654476	0.999627	114.7139			Illumina NovaSeq S4	91.36	0.83	1.886	50	0.056	57.25	40	1	0	1
BIF3	UW_DM_BIF3_1	DMB	JALCON000000000.1	SRX12654472		112.6135	d__Bacteria;p__Actinobacteriota;c__Actinomycetia;o__Actinomycetales;f__Bifidobacteriaceae;g__Bifidobacterium;s__Bifidobacterium crudilactis	GCF_000738005.1	Illumina NovaSeq S4	91.12	0.76	2.104	74	0.037	57.78	43	0	0	0
BIF4	UW_DM_BIF4_1	DMB	JALCOX000000000.1	SRX12654474		111.8635	d__Bacteria;p__Actinobacteriota;c__Actinomycetia;o__Actinomycetales;f__Bifidobacteriaceae;g__;s__	NA	Illumina NovaSeq S4	89.58	0	1.703	71	0.028	58.33	41	1	0	0
	UW_DM_BIF4_2	DMB	JALCOM000000000.1	SRX12654472	0.999837	99.6889			Illumina NovaSeq S4	77.5	0	1.570	70	0.027	58.15	35	0	0	0
BURK1	UW_DM_BURK1_1	DMB	JAKVPB000000000.1	SRX12687775		128.9295	d__Bacteria;p__Proteobacteria;c__Gammaproteobacteria;o__Burkholderiales;f__Burkholderiaceae;g__Mesosutterella;s__Mesosutterella multiformis	GCF_003402575.1	PacBio Sequel II	97.5	0	1.923	1	1.923	57.22	55	6	6	6
	UW_DM_BURK1_2	DMB	JAKVPH000000000.1	SRX12687771, SRX12687772	0.999958	128.9240			PacBio Sequel II	97.5	0	1.918	1	1.918	57.23	55	6	6	6
CLOS1	UW_DM_CLOS1_1	DMB	JALCQP000000000.1	SRX12654475		123.9345	d__Bacteria;p__Firmicutes_A;c__Clostridia;o__Clostridiales;f__Clostridiaceae;g__Clostridium_B;s__Clostridium_B tyrobutyricum	GCF_000359585.1	Illumina NovaSeq S4	98.19	0.23	2.862	38	0.148	30.79	59	3	1	0
	UW_DM_CLOS1_2	DMB	JALCRD000000000.1	SRX12654480	0.999992	123.9345			Illumina NovaSeq S4	98.19	0.23	2.913	39	0.148	30.74	59	3	1	0
	UW_DM_CLOS1_3	DMB	JALCQB000000000.1	SRX12654477	0.999997	123.9345			Illumina NovaSeq S4	98.19	0.23	2.873	38	0.148	30.79	59	3	1	0
	UW_DM_CLOS1_4	DMB	JALCPK000000000.1	SRX12654476	0.999996	105.6595			Illumina NovaSeq S4	79.8	0	2.520	34	0.148	30.73	53	3	1	0
CLOS2	UW_DM_CLOS2_1	DMB	JALCPJ000000000.1	SRX12654476		123.1268	d__Bacteria;p__Firmicutes_A;c__Clostridia;o__Clostridiales;f__Clostridiaceae;g__Clostridium_B;s__Clostridium_B luticellarii	GCF_002995845.1	Illumina NovaSeq S4	99.14	0	3.126	75	0.062	35.3	61	3	0	0
	UW_DM_CLOS2_2	DMB	JALCQA000000000.1	SRX12654477	0.999892	122.7914			Illumina NovaSeq S4	98.45	0	2.960	62	0.074	35.24	55	2	0	0
	UW_DM_CLOS2_3	DMB	JALCQO000000000.1	SRX12654475	0.999107	122.0420			Illumina NovaSeq S4	98.45	0	2.903	76	0.052	35.19	63	1	0	0
	UW_DM_CLOS2_4	DMB	JALCRC000000000.1	SRX12654480	0.999090	121.9077			Illumina NovaSeq S4	98.45	0.69	3.048	78	0.057	35.14	62	1	0	0
DIAL1	UW_DM_DIAL1_1	DMB	JAKVPC000000000.1	SRX12687775		124.9117	d__Bacteria;p__Firmicutes_C;c__Negativicutes;o__Veillonellales;f__Dialisteraceae;g__Dialister;s__Dialister hominis	GCF_007164725.1	PacBio Sequel II	94.21	0.02	1.867	2	1.382	47.9	49	3	3	3
	UW_DM_DIAL1_2	DMB	JAKVPI000000000.1	SRX12687771, SRX12687772	0.998770	124.1539			PacBio Sequel II	92.95	0.02	1.742	1	1.742	47.72	45	4	3	4
ENTER1	UW_DM_ENTER1_1	DMB	JALCOL000000000.1	SRX12654472		122.2348	d__Bacteria;p__Firmicutes;c__Bacilli;o__Lactobacillales;f__Enterococcaceae;g__;s__	NA	Illumina NovaSeq S4	96.69	1.66	2.371	26	0.168	47.22	59	1	1	0
	UW_DM_ENTER1_2	DMB	JALCOW000000000.1	SRX12654474	0.999971	107.0788			Illumina NovaSeq S4	81.22	0.55	1.874	21	0.168	47.9	45	1	1	0
FLAV1	UW_DM_FLAV1_1	Inoc.	JALCLE000000000.1	SRR19542533, SRR19542534		124.5230	d__Bacteria;p__Bacteroidota;c__Bacteroidia;o__Flavobacteriales;f__PHOS-HE28;g__PHOS-HE28;s__	NA	PacBio Sequel II	96.73	0.18	3.670	9	0.595	56.92	41	0	1	1
LAC1	UW_DM_LAC1_1	DMB	JAKVPJ000000000.1	SRX12687771, SRX12687772		122.4816	d__Bacteria;p__Firmicutes;c__Bacilli;o__Lactobacillales;f__Lactobacillaceae;g__Lactobacillus;s__	NA	PacBio Sequel II	92.18	0.52	1.430	2	1.290	42.69	56	4	4	4
	UW_DM_LAC1_2	DMB	JAKVPD000000000.1	SRX12687775	0.998540	121.7920			PacBio Sequel II	91.39	0.52	1.351	1	1.351	42.74	57	4	4	4
	UW_DM_LAC1_3	DMB	JALCOK000000000.1	SRX12654472	0.999299	113.7841			Illumina NovaSeq S4	89.56	0.26	1.182	23	0.074	42.95	18	0	0	0
	UW_DM_LAC1_4	DMB	JALCOV000000000.1	SRX12654474	0.999136	112.4220			Illumina NovaSeq S4	88.27	0.26	1.190	26	0.072	42.88	19	0	0	0
	UW_DM_LAC1_5	DMB	JALCPZ000000000.1	SRX12654477	0.998351	107.5929			Illumina NovaSeq S4	85.22	0.65	1.164	44	0.030	42.75	19	0	0	0
	UW_DM_LAC1_6	DMB	JALCPI000000000.1	SRX12654476	0.998014	105.8329			Illumina NovaSeq S4	83.59	0.59	1.208	45	0.030	42.76	20	0	0	0
LAC2	UW_DM_LAC2_1	DMB	JALCPY000000000.1	SRX12654477		109.0679	d__Bacteria;p__Firmicutes;c__Bacilli;o__Lactobacillales;f__Lactobacillaceae;g__Lactobacillus;s__Lactobacillus delbrueckii	GCF_001433875.1	Illumina NovaSeq S4	86.28	0	1.509	53	0.036	50.72	57	3	5	1
	UW_DM_LAC2_2	DMB	JALCPH000000000.1	SRX12654476	0.999965	108.7292			Illumina NovaSeq S4	85.63	0	1.438	47	0.041	50.88	54	3	5	1
LAC3	UW_DM_LAC3_1	DMB	JALCOU000000000.1	SRX12654474		98.8007	d__Bacteria;p__Firmicutes;c__Bacilli;o__Lactobacillales;f__Lactobacillaceae;g__Lactobacillus;s__Lactobacillus amylovorus	GCF_002706375.1	Illumina NovaSeq S4	77.52	0.08	1.247	53	0.028	38.13	29	0	0	0
LACCAS1	UW_DM_LACCAS1_1	DMB	JALCPX000000000.1	SRX12654477		121.7694	d__Bacteria;p__Firmicutes;c__Bacilli;o__Lactobacillales;f__Lactobacillaceae;g__Lacticaseibacillus;s__Lacticaseibacillus sp900540605	GCA_900540605.1	Illumina NovaSeq S4	97.08	0.52	2.326	32	0.097	53.4	44	1	1	0
	UW_DM_LACCAS1_2	DMB	JALCOJ000000000.1	SRX12654472	0.999925	119.7601			Illumina NovaSeq S4	95.54	0.7	2.305	39	0.073	53.49	32	1	1	1
	UW_DM_LACCAS1_3	DMB	JALCPG000000000.1	SRX12654476	0.999962	118.9627			Illumina NovaSeq S4	94.24	0.52	2.248	25	0.099	53.41	24	2	1	0
	UW_DM_LACCAS1_4	DMB	JALCRB000000000.1	SRX12654480	0.999897	115.7615			Illumina NovaSeq S4	93.51	0.52	2.160	82	0.032	53.58	18	1	0	0
	UW_DM_LACCAS1_5	DMB	JALCQN000000000.1	SRX12654475	0.999896	114.7656			Illumina NovaSeq S4	92.21	0.52	2.092	76	0.036	53.74	27	3	1	0
	UW_DM_LACCAS1_6	DMB	JALCOT000000000.1	SRX12654474	0.999964	103.8451			Illumina NovaSeq S4	81.94	0.52	1.920	84	0.027	53.86	23	1	0	0
LACCAS2	UW_DM_LACCAS2_1	DMB	JALCOI000000000.1	SRX12654472		118.8767	d__Bacteria;p__Firmicutes;c__Bacilli;o__Lactobacillales;f__Lactobacillaceae;g__Lacticaseibacillus;s__Lacticaseibacillus rhamnosus	GCA_000615245.1	Illumina NovaSeq S4	95.13	1.63	2.831	74	0.071	46.56	21	1	2	0
LACCAS3	UW_DM_LACCAS3_1	DMB	JALCQM000000000.1	SRX12654475		114.9943	d__Bacteria;p__Firmicutes;c__Bacilli;o__Lactobacillales;f__Lactobacillaceae;g__Lacticaseibacillus;s__	NA	Illumina NovaSeq S4	91.97	1.31	2.771	73	0.054	52.71	27	3	0	0
	UW_DM_LACCAS3_2	DMB	JALCRA000000000.1	SRX12654480	0.999829	112.0990			Illumina NovaSeq S4	89.18	1.31	2.661	66	0.052	52.96	45	3	0	0
LACCAS4	UW_DM_LACCAS4_1	DMB	JALCPF000000000.1	SRX12654476		95.9860	d__Bacteria;p__Firmicutes;c__Bacilli;o__Lactobacillales;f__Lactobacillaceae;g__Lacticaseibacillus;s__Lacticaseibacillus paracasei	GCF_000829035.1	Illumina NovaSeq S4	75.18	0	1.611	83	0.023	46.77	44	1	0	0
LCO1	UW_DM_LCO1_1	Inoc.	JALCKY000000000.1	SRR19542531, SRR19542532		118.3603	d__Bacteria;p__Firmicutes_A;c__Clostridia;o__Lachnospirales;f__Lachnospiraceae;g__;s__	NA	PacBio Sequel II	91.14	1.32	3.027	11	0.376	51.98	47	2	2	2
	UW_DM_LCO1_2	Inoc.	JALCLD000000000.1	SRR19542533, SRR19542534	0.994960	106.9934			PacBio Sequel II	78.48	0.21	2.047	4	0.528	52.45	33	1	1	1
LENLAC1	UW_DM_LENLAC1_1	DMB	JALCQL000000000.1	SRX12654475		121.1830	d__Bacteria;p__Firmicutes;c__Bacilli;o__Lactobacillales;f__Lactobacillaceae;g__Lentilactobacillus;s__Lentilactobacillus hilgardii	GCF_000159315.1	Illumina NovaSeq S4	96.75	0.31	2.943	54	0.082	39.99	45	3	0	0
	UW_DM_LENLAC1_2	DMB	JALCPE000000000.1	SRX12654476	0.999777	113.1572			Illumina NovaSeq S4	88.63	0.08	1.421	27	0.079	39.34	33	2	0	0
	UW_DM_LENLAC1_3	DMB	JALCQZ000000000.1	SRX12654480	0.999879	113.1317			Illumina NovaSeq S4	88.56	0	1.374	25	0.082	39.3	35	1	0	0
	UW_DM_LENLAC1_4	DMB	JALCPW000000000.1	SRX12654477	0.999860	102.4262			Illumina NovaSeq S4	76.75	0	1.009	15	0.136	39.15	12	0	0	0
LEUC1	UW_DM_LEUC1_1	DMB	JALCOH000000000.1	SRX12654472		120.7671	d__Bacteria;p__Firmicutes;c__Bacilli;o__Lactobacillales;f__Lactobacillaceae;g__Leuconostoc;s__Leuconostoc mesenteroides	GCF_000014445.1	Illumina NovaSeq S4	91.4	0.53	1.522	4	0.840	37.62	28	1	0	0
	UW_DM_LEUC1_2	DMB	JALCOS000000000.1	SRX12654474	0.999988	120.7671			Illumina NovaSeq S4	91.4	0.53	1.525	5	0.840	37.62	28	1	0	0
	UW_DM_LEUC1_3	DMB	JAKVPK000000000.1	SRX12687771, SRX12687772	0.999972	107.4873			PacBio Sequel II	80.29	1.06	1.632	11	0.350	37.69	65	2	2	2
LIGLAC1	UW_DM_LIGLAC1_1	DMB	JALCPD000000000.1	SRX12654476		110.5664	d__Bacteria;p__Firmicutes;c__Bacilli;o__Lactobacillales;f__Lactobacillaceae;g__Ligilactobacillus;s__Ligilactobacillus acidipiscis	GCF_001435755.1	Illumina NovaSeq S4	89.01	1.83	1.563	66	0.031	39.32	27	1	0	0
	UW_DM_LIGLAC1_2	DMB	JALCPV000000000.1	SRX12654477	0.999639	110.1389			Illumina NovaSeq S4	88.48	1.31	1.526	67	0.029	39.19	13	1	0	0
LIMLAC1	UW_DM_LIMLAC1_1	DMB	JAKVPL000000000.1	SRX12687772, SRX12687771		130.6667	d__Bacteria;p__Firmicutes;c__Bacilli;o__Lactobacillales;f__Lactobacillaceae;g__Limosilactobacillus;s__	NA	PacBio Sequel II	98.27	1.09	2.335	1	2.335	52.19	71	6	6	6
	UW_DM_LIMLAC1_2	DMB	JAKVPE000000000.1	SRX12687775	0.999996	130.6667			PacBio Sequel II	98.27	1.09	2.335	1	2.335	52.19	71	6	6	6
LIMLAC2	UW_DM_LIMLAC2_1	DMB	JAKVPM000000000.1	SRX12687772, SRX12687771		130.4626	d__Bacteria;p__Firmicutes;c__Bacilli;o__Lactobacillales;f__Lactobacillaceae;g__Limosilactobacillus;s__Limosilactobacillus oris	GCF_001434465.1	PacBio Sequel II	98.9	0	2.044	1	2.044	49.58	62	5	5	5
	UW_DM_LIMLAC2_2	DMB	JAKVPF000000000.1	SRX12687775	1.000000	130.4626			PacBio Sequel II	98.9	0	2.044	1	2.044	49.58	62	5	5	5
	UW_DM_LIMLAC2_3	DMB	JALCQK000000000.1	SRX12654475	0.999998	123.0035			Illumina NovaSeq S4	97.79	0	1.907	22	0.110	49.92	28	1	1	0
	UW_DM_LIMLAC2_4	DMB	JALCQY000000000.1	SRX12654480	0.999984	106.9135			Illumina NovaSeq S4	81.7	0	1.286	18	0.110	49.19	29	1	0	0
LIMLAC3	UW_DM_LIMLAC3_1	DMB	JALCQJ000000000.1	SRX12654475		117.6852	d__Bacteria;p__Firmicutes;c__Bacilli;o__Lactobacillales;f__Lactobacillaceae;g__Limosilactobacillus;s__Limosilactobacillus timonensis	GCF_900240275.1	Illumina NovaSeq S4	94.02	0	1.286	32	0.054	47.82	19	0	0	0
	UW_DM_LIMLAC3_2	DMB	JALCPU000000000.1	SRX12654477	1.000000	117.5831			Illumina NovaSeq S4	94.02	0	1.292	35	0.051	47.59	18	0	0	0
	UW_DM_LIMLAC3_3	DMB	JALCPC000000000.1	SRX12654476	1.000000	115.9531			Illumina NovaSeq S4	92.39	0	1.261	33	0.051	47.58	18	0	0	0
	UW_DM_LIMLAC3_4	DMB	JALCQX000000000.1	SRX12654480	0.999991	115.5152			Illumina NovaSeq S4	91.85	0	1.216	30	0.054	47.71	16	0	0	0
LIMLAC4	UW_DM_LIMLAC4_1	DMB	JALCQI000000000.1	SRX12654475		114.5738	d__Bacteria;p__Firmicutes;c__Bacilli;o__Lactobacillales;f__Lactobacillaceae;g__Limosilactobacillus;s__	NA	Illumina NovaSeq S4	91.37	0	1.531	42	0.044	41.25	30	3	0	1
	UW_DM_LIMLAC4_2	DMB	JALCQW000000000.1	SRX12654480	0.999945	112.9835			Illumina NovaSeq S4	90.42	0	1.522	58	0.032	40.99	33	3	0	2
LIQLAC	UW_DM_LIQLAC1_1	DMB	JALCPB000000000.1	SRX12654476		125.1084	d__Bacteria;p__Firmicutes;c__Bacilli;o__Lactobacillales;f__Lactobacillaceae;g__Liquorilactobacillus;s__Liquorilactobacillus nagelii	GCF_001434225.1	Illumina NovaSeq S4	99.48	0.13	2.380	22	0.137	36.57	24	2	1	0
	UW_DM_LIQLAC1_2	DMB	JALCPT000000000.1	SRX12654477	0.999945	124.4405			Illumina NovaSeq S4	99.48	0.13	2.518	40	0.101	36.57	23	1	1	0
MEG1	UW_DM_MEG1_1	DMB	JAKVPG000000000.1	SRX12687775		129.5279	d__Bacteria;p__Firmicutes_C;c__Negativicutes;o__Veillonellales;f__Megasphaeraceae;g__Megasphaera;s__Megasphaera sp000417505	GCF_000417505.1	PacBio Sequel II	97.63	0	2.386	1	2.386	53.63	64	7	7	7
	UW_DM_MEG1_2	DMB	JAKVPN000000000.1	SRX12687772, SRX12687771	1.000000	129.5279			PacBio Sequel II	97.63	0	2.386	1	2.386	53.63	64	7	7	7
	UW_DM_MEG1_3	DMB	JALCOG000000000.1	SRX12654472	0.999984	122.3850			Illumina NovaSeq S4	97.41	0	2.141	33	0.098	54.24	59	2	0	0
	UW_DM_MEG1_4	DMB	JALCOR000000000.1	SRX12654474	0.999956	122.3849			Illumina NovaSeq S4	97.41	0	2.137	33	0.098	54.28	59	2	0	0
PREV1	UW_DM_PREV1_1	Inoc.	JALCLC000000000.1	SRR19542533, SRR19542534		114.1849	d__Bacteria;p__Bacteroidota;c__Bacteroidia;o__Bacteroidales;f__Bacteroidaceae;g__Prevotella;s__	NA	PacBio Sequel II	89.07	0	2.465	18	0.166	37.89	53	4	4	4
PSEUD1	UW_DM_PSEUD1_1	Inoc.	JALCKX000000000.1	SRR19542531, SRR19542532		117.1056	d__Bacteria;p__Proteobacteria;c__Gammaproteobacteria;o__Pseudomonadales;f__Pseudomonadaceae;g__Pseudomonas_E;s__Pseudomonas_E veronii	GCF_001439695.1	PacBio Sequel II	91.21	2.49	6.322	21	0.423	61.35	63	4	4	4
SACCH1	UW_DM_SACCH1_1	Inoc.	JALCKW000000000.1	SRR19542531, SRR19542532		111.6234	d__Bacteria;p__Firmicutes_A;c__Clostridia;o__Saccharofermentanales;f__Saccharofermentanaceae;g__Saccharofermentans;s__	NA	PacBio Sequel II	86.5	0.71	1.640	13	0.142	46.48	36	1	0	1
SCHLAC1	UW_DM_SCHLAC1_1	DMB	JALCOF000000000.1	SRX12654472		121.0337	d__Bacteria;p__Firmicutes;c__Bacilli;o__Lactobacillales;f__Lactobacillaceae;g__Schleiferilactobacillus;s__Schleiferilactobacillus perolens	GCF_001435585.1	Illumina NovaSeq S4	97.77	1.05	2.820	76	0.057	49.2	54	1	0	0
	UW_DM_SCHLAC1_2	DMB	JALCOQ000000000.1	SRX12654474	0.999809	120.1456			Illumina NovaSeq S4	96.86	0.52	2.885	79	0.051	49.21	42	1	0	1
SPHING1	UW_DM_SPHING1_1	Inoc.	JALCLB000000000.1	SRR19542533, SRR19542534		114.1032	d__Bacteria;p__Proteobacteria;c__Alphaproteobacteria;o__Sphingomonadales;f__Sphingomonadaceae;g__Sphingobium;s__	NA	PacBio Sequel II	86.54	0.78	3.532	10	0.615	62.87	46	1	1	1
SPOR1	UW_DM_SPOR1_1	DMB	JALCOE000000000.1	SRX12654472		110.2120	d__Bacteria;p__Firmicutes;c__Bacilli;o__Bacillales_G;f__Sporolactobacillaceae;g__Sporolactobacillus;s__Sporolactobacillus sp900543345	GCA_900543345.1	Illumina NovaSeq S4	88.48	0	2.806	95	0.035	49.49	28	2	1	0
XAN1	UW_DM_XAN1_1	Inoc.	JALCLA000000000.1	SRR19542533, SRR19542533		124.1187	d__Bacteria;p__Proteobacteria;c__Gammaproteobacteria;o__Xanthomonadales;f__Xanthomonadaceae;g__Chiayiivirga;s__	NA	PacBio Sequel II	94.97	2.71	3.678	9	0.762	66.87	52	2	2	2
XAN2	UW_DM_XAN2_1	Inoc.	JALCKV000000000.1	SRR19542531, SRR19542532		113.2098	d__Bacteria;p__Proteobacteria;c__Gammaproteobacteria;o__Xanthomonadales;f__Xanthomonadaceae;g__Chiayiivirga;s__	NA	PacBio Sequel II	82.96	0.86	3.747	2	1.891	66.94	46	2	2	2

a Strain code abbreviations are as follows: ACET, *Acetobacter*; ACT, *Actinomycetaceae*; ACUT, *Acutalibacteraceae*; ANA, *Anaerotignaceae*; ATO, *Atopobiaceae*; BACIL, *Bacilli*; BACTE, Bacteroidales; BIF, *Bifidobacteriaceae*; BURK, *Burkholderiaceae*; CLOS, *Clostridium*; DIAL, *Dialisteraceae*; ENTER, *Enterococcaceae*; FLAV, *Flavobacteriales*; LAC, *Lactobacillus*; LACCAS, *Lacticaseibacillus*; LCO, *Lachnospiraceae*; LENLAC, *Lentilactobacillus*; LEUC, *Leuconostoc*; LIGLAC, *Ligilactobacillus*; LIMLAC, *Limosilactobacillus*; LIQLAC, *Liquorilactobacillus*; MEG, *Megasphaera*; PREV, *Prevotella*; PSEUD, *Pseudomonadaceae*; SACCH, *Saccharofermentans*; SCHLAC, *Schleiferilactobacillus*; SPHING, *Sphingobium*; SPOR, *Sporolactobacillus*; XAN, *Xanthomonadaceae*.

b Strain name assigned to each reported MAG. The abbreviation UW_DM stands for University of Wisconsin dairy manure bioreactor. The strain name followed by “_1” denotes the representative MAG, as selected using dRep (15) for each cluster; other MAGs in the same cluster use the same strain name, followed by a number in increasing order, and are sorted by dRep score.

c The sample source of each MAG is indicated either by DMB (dairy manure bioreactor) or Inoc. (inoculum).

d The following are the number of filtered raw reads corresponding to each SRA accession number: SRR19542531, 111,403; SRR19542532, 152,018; SRR19542533, 134,737; SRR19542534, 259,819; SRX12654472, 112,981,324; SRX12654474, 76,248,910; SRX12654475, 99,005,980; SRX12654476, 111,069,392; SRX12654477, 105,196,124; SRX12654480, 95,275,964; SRX12687771, 110,527; SRX12687772, 119,805; SRX12687775, 191,571.

e ANIm, average nucleotide identity between the representative MAG and other MAGs included in the same cluster calculated using dRep (15).

f dRep scoring calculation: $\text{A}\;\times \;\mathrm{completeness}\;-\;\text{B}\;\times \;\mathrm{contamination}\;+\;\text{C}\;\times \;\left[\mathrm{contamination}\;\times \;\left(\mathrm{strain heterogeneity}/100\right)\right]\;+\;\text{D}\;\times \;\log\left(N_{50}\right)\;+\;\text{E}\;\times \;\log\left(\mathrm{genome size}\right)\;+\;\text{F}\;\times \;\left(\mathrm{centrality}\;-\;\text{S}\_\mathrm{ANI}\right)$ where A through F were weighted with the values 1, 0.5, 1, 5, 0, and 1, respectively.

g Classifications made using the Genome Taxonomy Database Toolkit (GTDB-Tk) (17).

h GenBank accession number of the reference genome in GTDB-Tk that is closest to the representative MAG, determined using RAxML-NG (18); NA (not applicable) indicates MAGs without a closely matched reference genome when using the default minimum alignment fraction of 0.65.

### Data availability.

The raw metagenomic sequence data and the refined MAGs are available at NCBI GenBank under BioProject accession number PRJNA768492. All information on the library construction and sequencing can be found at https://gold.jgi.doe.gov/study?id=Gs0150020 using the JGI GOLD study ID Gs0150020. All custom scripts are available on GitHub (https://github.com/GLBRC/metagenome_analysis).
